# 3D Gold Nanowire Networks with Tailorable Surface Wetting State: From Rose‐Petal Effect to Super‐Hydrophilicity

**DOI:** 10.1002/smll.202411971

**Published:** 2025-04-14

**Authors:** Mohan Li, Henning Bonart, Daniel Zellner, Maria Eugenia Toimil‐Molares

**Affiliations:** ^1^ Materials Research Department GSI Helmholtzzentrum für Schwerionenforschung 64291 Darmstadt Germany; ^2^ Department of Materials Science Technical University of Darmstadt 64287 Darmstadt Germany; ^3^ Institute for Nano‐ and Microfluidics Technical University of Darmstadt 64287 Darmstadt Germany

**Keywords:** ion‐track nanotechnology, nanostructure, nanowire network, rose‐petal effect, wetting state

## Abstract

This study demonstrates the different wetting states that can be achieved by varying the diameter and density of nanowires in free‐standing 3D gold nanowire networks. This network structure consists of nanowires oriented at 45° to the horizontal plane and interconnected from four different directions. Sessile drop measurements on these tailored nanostructured films show a transition from hydrophilic to hydrophobic behavior as porosity increases from 20% to 98%. With tailored porosity from 60% to 80%, this nanostructure can exhibit super‐hydrophilicity. In addition, the highly porous (>90%) hydrophobic structures exhibit the rose‐petal effect, where water droplets remain pinned to the surface. These novel results demonstrate the capability to precisely control surface wetting behavior through intricate designs of nanostructures, which are crucial for a wide range of applications, including liquid transport, microfluidic devices, and sensors.

## Graphical abstract

This study demonstrates that different wetting states can be achieved by varying the diameter and density of nanowires in free‐standing 3D gold nanowire networks. Sessile drop measurements on these tailored nanostructured films showed a transition from hydrophilic to hydrophobic (rose‐petal effect) behavior as porosity increased from 20% to 98%.

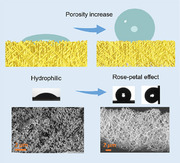

## 1 Introduction

Wetting phenomena on solid surfaces, particularly when considering nanostructured surfaces, display intriguing and complex interactions with different kinds of liquids captivating the attention of researchers in various scientific fields.^[^ ^1^, ^2^, ^3^, ^4^, ^5^, ^6^, ^7^ ^]^ It has been demonstrated that the surface wetting state is impacted by the unique characteristics of nanostructures present on the surfaces, including nanoparticles, nanowires, and other nanoscale features,^[^ ^4^, ^6^, ^8^, ^9^, ^10^, ^11^, ^12^ ^]^ and in particular by parameters such as surface‐area‐to‐volume ratio, intricate surface roughness, and the surface chemical composition.^[^ ^12^, ^13^, ^14^, ^15^, ^16^, ^17^ ^]^ For example, it has been shown that artificial nanostructures create superhydrophobic surfaces with water droplets forming nearly spherical shapes with high contact angles due to reduced solid‐liquid contact.^[^ ^18^, ^19^, ^20^, ^21^ ^]^ In nature, anisotropic wetting has been widely observed on various biological surfaces, including plant leaves, insect wings, and animal skins, which exhibit directional wetting properties that have evolved to serve specific functions.^[^ ^22^, ^23^, ^24^, ^25^ ^]^ In nature, we can also find intriguing complementary effects such as the lotus and the rose petal effects. The lotus effect shows a hydrophobic behavior, where the droplet will roll off the tilted surface, and shows very low surface adhesion and low contact angle hysteresis.^[^ ^6^, ^26^, ^27^, ^28^, ^29^ ^]^ On the other hand, the rose petal surface exhibits a high contact angle with high adhesion. The droplet is thus pinned on the surface and forms a high contact angle hysteresis, which is a special wetting phenomenon.^[^ ^30^, ^31^ ^]^ These effects are both the result of special surface structures, which exhibit specific roughness in both the micrometer and nanometer ranges.^[^ ^32^, ^33^, ^34^, ^35^, ^36^ ^]^

With proper designs, synthetic structures can mimic natural hydrophobic behavior like the one displayed by lotus leaves or insect wings, which effectively repel water,^[^ ^37^, ^38^ ^]^ or they can, in turn, enhance the surface wettability, such as hierarchical surface patterns, for applications like microfluidic devices, where precise control of liquid flow is critical.^[^ ^8^, ^39^, ^40^ ^]^

The contact angle on a surface is the angle formed at the three‐phase contact line, where a liquid, a solid, and a gas phase (usually air) meet. The contact angle of a droplet on a solid is given by the Young's equation:

(1) $$\cos\left(\theta_{0}\right)=\left(\gamma_{S}-\gamma_{SL}\right)/\gamma_{L}$$

where *γ_S_* is the surface energy of the solid, *γ_L_* is the surface energy of the liquid, and *γ_SL_* is the energy of the solid‐liquid interface.^[^ ^41^ ^]^

Two classical models describe a deposited water droplet sitting on a rough surface: the Wenzel^[^ ^42^ ^]^ and Cassie–Baxter^[^ ^43^ ^]^ models, providing a theoretical framework to explain how surface roughness influences wetting. The Wenzel theory describes a chemically homogeneous surface where the liquid can completely wet the surface, while the Cassie–Baxter theory can describe a chemically heterogeneous surface,^[^ ^31^ ^]^ which has been be further developed and applied on a single‐phased rough surface with texture, the surface is only partially wetted, with air pockets trapped in the asperities.^[^ ^44^ ^]^ However, different wetting states can coincide on the same heterogeneous surface and existing theories only offer partial solutions to the continuously evolving field of complex surfaces.^[^ ^45^, ^46^ ^]^ By integrating various hydrophilic and hydrophobic nanostructures, it is possible to achieve a multitude of unique functionalities. This innovative research can yield surfaces with precisely tailored wettability, such as enhanced self‐cleaning capabilities, superior anti‐fouling properties, and optimized fluid transport mechanisms.^[^ ^25^ ^]^

Exploring new combinations of nanostructures and surface chemical compositions can yield surfaces with unique and enhanced functionalities. In this work, we investigate the wettability of tailored 3D porous structures in the form of freestanding nanowire networks with controlled porosity. These nanostructures are created by the electrodeposition of gold (Au) nanowires in etched ion‐track templates, which we will refer to as the nanowire network (NWNW) structure^[^ ^47^ ^]^ Gold was selected for this study, due to its chemical stability.^[^ ^45^ ^]^ Despite the hydrophilic nature of bulk gold,^[^ ^48^ ^]^ our porous structures exhibit various wetting states depending on their geometry. This investigation explores the intricate design of porous nanostructures and the macroscopic wetting behavior on nanowire networks (NWNWs), contributing to our understanding of how liquids interact with structured surfaces, and to the broadening of manufacturing methods for biomimetic materials.

## 2 Results and Discussion

### 2.1 Morphology and Structure

**Figure 1:**
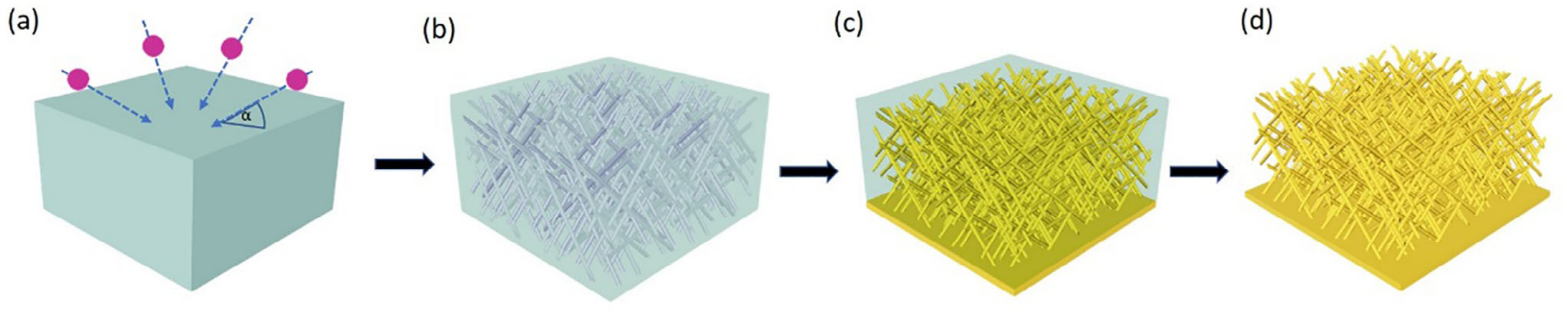
Schematic of nanowire network fabrication: a) a PC polymer foil is irradiated with swift heavy ions (indicated by arrows) from four directions, in each case at an angle α = 45° to the foil surface, b) chemical etching of the generated ion tracks leads to the formation of a 3D nanochannel network, c) fabrication of Au substrate layer and electrodeposition of Au inside the nanochannels, and d) removal of the polymer matrix to obtain a freestanding 3D nanowire network. Duplicate from M. Li, et al., RSC Advances, 2023.^[^ ^47^ ^]^

Free‐standing 3D Au NWNWs were synthesized by electrodeposition in ion track‐etched polycarbonate (PC) templates (**Figure** **1**). We fabricated two series of free‐standing Au nanowire networks, with systematic variations of their geometrical parameters, namely nanowire diameter and nanowire density (see details in Experimental Section and Figure S1, Supporting Information).

**Figure 2:**
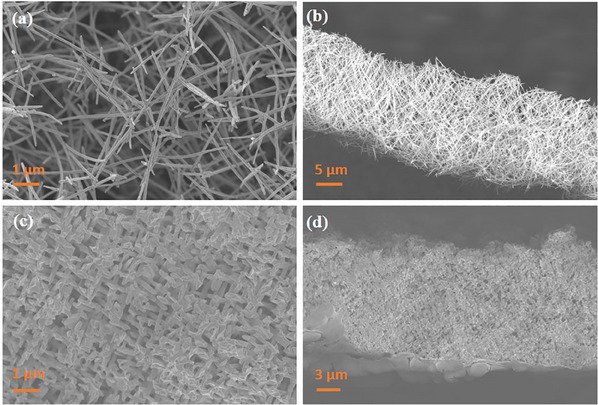
Representative SEM images of a low‐density Au NWNW with 100 nm wire diameter with an estimated 95% porosity a,b), and one high‐density Au NWNW with 200 nm wire diameter with an estimated 28% porosity c,d). (a)/(c) and (b)/(d) display top‐ and side views, respectively.

One series of “low‐density” NWNWs with a density (2.3±0.3) × 10^8^ wires cm^−2^ and nanowire diameters ranging between 60 and 200 nm, and another series of “high‐density” NWNWs with a density (1.3±0.1) × 10^9^ wires cm^−2^ and wire diameters between 40 and 200 nm. The nanowire density was estimated from scanning electron microscopy (SEM) analysis of the corresponding PC thin films used as templates (see Figure S2, Supporting Information). The minimum nanowire diameter value was selected for each density to guarantee the interconnectivity and stability of the nanowire networks. Especially for the low‐density series, nanowires with diameters smaller than 60 nm provided too few interconnection points resulting in unstable network structures. **Figure** **2** shows the top and side views of two representative samples from each series, (a,b) low‐density and (c,d) high‐density. With the variations in wire density and wire diameter, we were able to tune the network structure porosity from 20% to 98%. A detailed calculation of the porosity values based on the measured nanowire diameter, network height, and nanowire density is available in the supporting information.

It is worth emphasizing that NWNWs with porosities as high as 98% can be achieved. Figure 2a,b shows a representative sample with 95% porosity, the structure of these highly porous Au NWNWs is still self‐supporting and mechanically stable. After the nanowires are freed from templates, since the highly porous NWNW is formed by thin wires and low wire density, the thinnest nanowires cannot maintain the original orientations, given by the template, and they rather bend appearing at times curved along their length (Figure 2b). With increasing wire density and wire diameter, the network structure becomes denser (Figure 2c,d), the nanowires can maintain their 45° orientation, and the average spacings between adjacent nanowires decrease with increasing wire density and diameter.

### 2.2 Contact Angle Measurement

**Figure 3:**
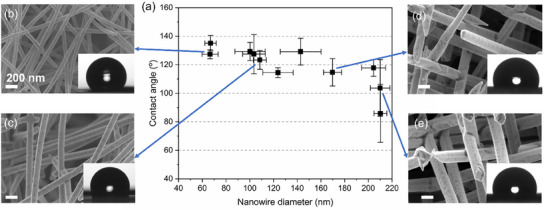
a) Contact angle measured for low‐density NWNWs with tailored nanowire diameter between 60 and 210 nm, and porosity ranging between 98% and 86%, respectively. SEM images show selected networks with nanowire diameters b) 60 nm, c) 100 nm, d) 160 nm, e) 210 nm, and the scale bar is 200 nm. The insets show the sessile drop on the corresponding sample surface. The error bars are given by the standard deviation of five contact angle measurements on various positions of the same sample.

**Figure** **3**a shows the contact angle measured for a series of low‐density Au NWNWs with wire diameters between 60 and 210 nm. For these samples, the porosity ranges between 98% and 86% (see details in Table S1, Supporting Information). The systematic increase in NW diameter is evident in the SEM images taken under the same magnification. While the thinner nanowires (Figure 3b,c) exhibit a bending geometry, the thicker ones (Figure 3d,e, diameter > 130 nm) appear straight, maintaining the original template channel orientation. The NWNWs with the smallest wire diameters (60–100 nm) exhibit an average contact angle of ≈130°. For thicker nanowire diameters (100–200 nm), the contact angle of the various NWNWs varies between 110° and 130°. For NWNWs with wire diameters above 200 nm a larger distribution of contact angles was observed including a transition to hydrophilic states.

**Figure 4:**
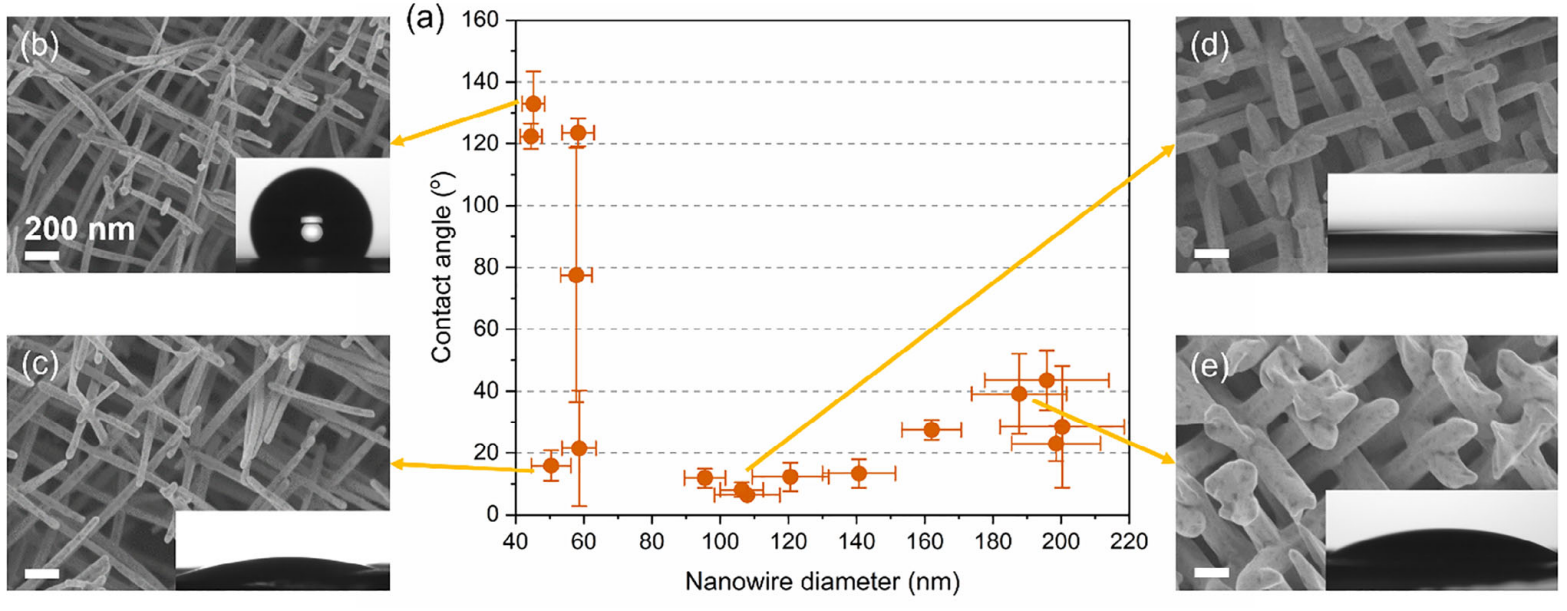
a) Contact angle measured for high‐density Au NWNWs with tailored nanowire diameter between 40 and 200 nm, and porosity ranging between 96% and 28%, respectively. SEM images show selected networks with nanowire diameters b) Ø 40 nm, c) Ø 60 nm, d) Ø 110 nm, e) Ø 180 nm, and the scale bar is 200 nm. The insets show the sessile drop on the corresponding sample surface. The error bars are given by the standard deviation of five contact angle measurements on various positions of the same sample.

**Figure** **4** displays the contact angle measurements of the high‐density series of Au NWNWs, with wire diameters ranging from 40 to 200 nm. For these samples, the porosity ranges between 96% and 28% (see details in Table S1, Supporting Information) The NWNWs of this series exhibit more crossing junctions than the low‐density NWNWs, so nanowires with diameters between 60 and 200 nm can still maintain their orientation. The sample with the thinnest nanowires (Ø ≈40 nm) displays bending wires and exhibits a rather high contact angle (120°–130°). When the wire diameter increases to ≈60 nm, we observed a transition stage, the contact angle ranging from 10° to 120°, this large deviation may be a result of the nanowire diameter distribution (≈10%) combined with the local randomly distributed spacing and pores between nanowires. When the wire diameter reaches ≈100 nm, the samples show super‐hydrophilicity, where the contact angle is smaller than 10°, and we observed a swift droplet spreading all over the surface. When the wire diameter further increases, the contact angle again rises and stabilizes at ≈40°, which is similar to a bulk gold surface.^[^ ^48^ ^]^

**Figure 5:**
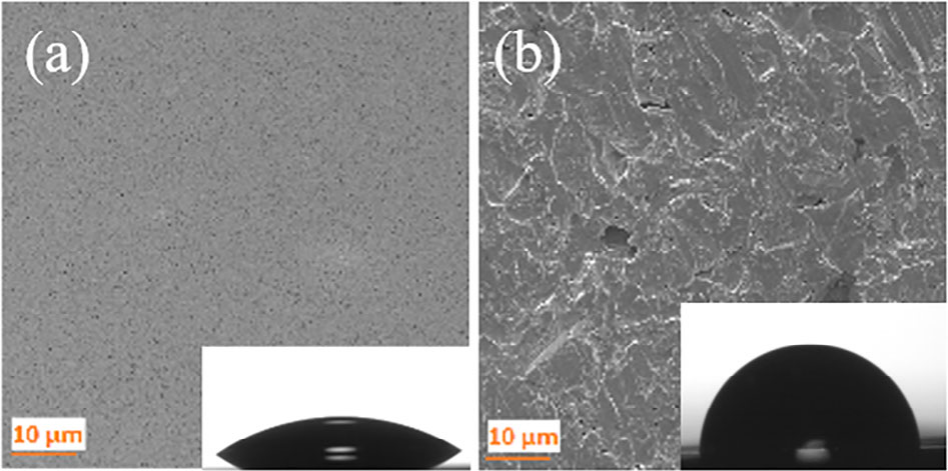
SEM images of a) Au sputtered silicon wafer, and b) Au substrate layer with insets of sessile drop for contact angle measurement.

For comparison, we fabricated two different Au planar reference surfaces, shown in **Figure** **5**. The first one is a ≈300 nm thick smooth Au layer sputtered on a Si wafer. The sputtered Au surface exhibits hydrophilicity with a contact angle of ≈40°, which represents the hydrophilic nature of Au, and is in agreement with previously reported values.^[^ ^48^, ^49^ ^]^ The second one is an electrodeposited Au thin film substrate layer, which adopted the roughness of the polycarbonate template surface. This Au thin film shows a higher contact angle (≈90°) attributed to the surface roughness. Obviously, the wettability of these two pure Au surfaces differs from the Au NWNW structure, supporting that the special wetting phenomena of NWNWs are mainly the result of this 3D nanostructure.

### 2.3 Comparison and Discussion

**Figure 6:**
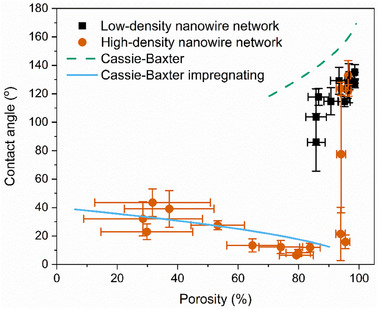
Measured contact angle values versus the nanowire network porosity visualizing the influence of the network porosity on wetting state transition. The corresponding values estimated with the models of Cassie‐Baxter (solid blue line) and Cassie‐Baxter impregnating (dash green line) are also shown.

To further study and understand the wettability transition by NWNW geometry variation, we calculated the porosity of each characterized network structure and plotted it against the measured contact angle, as shown in **Figure** **6**. The data indicates that the contact angle values show a well‐distinguishable trend independent of the nanowire density for most of the porosities. This strongly indicates that the structural porosity is one of the key parameters influencing the surface wetting state transition of the NWNWs.

For all low‐density NWNWs, the porosity ranges from 98% to 85%, and the contact angle decreases with decreasing porosity. All samples with porosity higher than 95%, exhibit high contact angles above 120° independent of the NW density. However, for porosities between 80% and 95%, the samples display mixed wetting behaviors, i.e., hydrophobic and hydrophilic in a sequential manner. This indicates that the water droplet is very sensitive to the surface microstructure, resulting in an unstable wetting behavior within this porosity range. Here, the contact angle varies widely between 120° and 10°. We define this phase as a transition state, in which the NWNW transits from hydrophobic to hydrophilic behavior. This transition regime can originate from the random distribution of ions, which is especially noticeable at high porosities, where nanowire density and diameter are smaller, leading to a larger distribution of interwire distances and subsequent local deviations from the original 45° nanowire orientation, which in turn affects the measured apparent contact angles. When the porosity is lower than 80%, the samples became super‐hydrophilic with contact angles smaller than 10°. Further decrease of the porosity to between 60% and 20% leads to a slight increase in the contact angle to ≈20°–50°.

This suggests that, as the porosity decreases, the interplay between the pore size distribution, surface structure, and drop contact line becomes increasingly significant in determining the wetting behavior. Following Bico et al. 2001,^[^ ^50^ ^]^ modeling the interfacial energies leads to a condition for a critical contact angle of imbibition. For a porous medium with interconnected channels or pores, this condition states that when the structure is invaded by liquids, the contact angle is smaller than 90°. Since the contact angle with smooth gold is ≈40°–60°, imbibition should theoretically occur for all the samples. However, as pointed out in the review article by Gambaryan–Roisman 2014,^[^ ^51^ ^]^ this model does not account for the influence of local substrate structures. The local structure governs the local position of the contact line. In addition, rather strong contact line pinning can lead to a metastable state of the drop on the substrate.

From our experimental results, we observed stable hydrophobic behavior for very high porosity (>90%) samples, while samples with porosities between 80% and 90% exhibit strong fluctuations in measured contact angles. We attribute this behavior to the fact that at this porosity range, where bending nanowires cannot maintain the original 45° wire orientation, local differences in nanowire orientation and distribution result in a variety of contact angles (Figure 2). We hypothesize that, for the hydrophobic samples, with very high porosity nanostructures, air may become trapped within the pores beneath the drop.^[^ ^52^ ^]^ Below a certain porosity (i.e., smaller local interwire distances), the drop is sometimes pinned to the structures therefore resulting in a hydrophilic contact angle.

Assuming that for the hydrophobic NWNWs, the drop sits on top of the bending nanowire structure with air trapped beneath, we can use the Cassie–Baxter equation,

(2) $$\cos\left(\theta^{*}\right)=-1+f_{s}(\cos\left(\theta_{Y})+1\right)$$

to calculate the apparent contact angle, where *f_s_* is the fractional solid surface area, and θ_*Y*_ is the contact angle for the smooth gold surface, obtaining the green dash line in Figure 6. It can be seen on the graph that the Cassie–Baxter model quantifies the trend of the low‐density samples very well. The discrepancy between the predicted and measured contact angle values is ≈20° for the hydrophobic samples. We attribute the systematic shift in contact angle values to the complex surface morphology of the designed NWNWs in contrast to the simplifications assumed made by the theoretical models.^[^ ^53^ ^]^

To model the imbibition of the drop for lower‐porosity NWNWs, we apply the impregnating Cassie–Baxter model,^[^ ^53^ ^]^

(3) $$\cos\left(\theta^{*}\right)=1+f_{s}\left(\cos\theta_{Y}-1\right)$$

obtaining the blue line in Figure 5, and demonstrating a very good agreement between the model prediction and the measured contact angles.

These results show a clear dependency between the effective porosity of the NWNWs and their wettability, with both high‐density and low‐density series. For very high‐porosity NWNWs, the droplet sits on top of the structure with trapped air, leading to a clear hydrophobic behavior (see Figure S4, Supporting Information), while for lower porosity NWNWs, imbibition is energetically favorable, with the liquid spreading across the rough hydrophilic surface driven by capillarity (details on the wetting process are shown in the Figures S5 and S6, Supporting Information). In addition, we found the hydrophobic samples do not exhibit a classical behavior as traditional hydrophobic surfaces, but there is a high adhesion between the droplet and the sample surface, so we performed additional measurements to examine the adhesion between the droplet and sample surface.

### 2.4 Droplet Sliding Measurement

**Figure 7:**
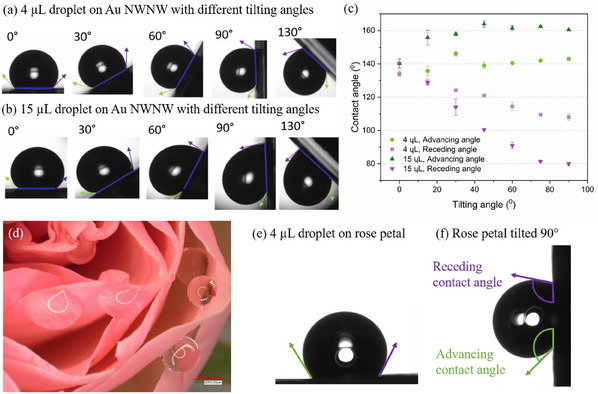
a) 4 µL and b) 15 µL water droplets sitting on Au NWNW surface with different tilting angles, c) plots of two‐volume water droplets advancing (green) and receding (purple) contact angles as a function of stage tilting angle, d) optical image of a rose with multiple droplets attached on the petals, e,f) images of a 4 µL water droplet on one rose petal surface: e) horizontal position, and f) tilted 90° with advancing (green) and receding (purple) angles.

The highly porous samples were mounted on a tilting stage, and the advancing and receding angles were measured. **Figure** **7** shows the representative results obtained for a high‐porosity low‐density NWNW with 60 nm diameter nanowires (more test results see Figure S7, Supporting Information). We employed two droplet sizes, 4 and 15 µL, for each sample, and they showed similar behavior, displayed in Figure 7a,b, respectively. The images evidence that with increasing tilting angle, the droplet advancing angle slightly increases while the receding angle largely decreases. Moreover, the measurements show a very high contact angle hysteresis, which is the difference between advancing and receding angles. We can observe a contact angle of 135° with a contact angle hysteresis of 35° (4 µL droplet) and 80° (15 µL droplet). This demonstrates an extremely high adhesion of the droplet to the substrate. Moreover, we performed the same measurement with a 4 µL droplet on a fresh rose petal, where we observed a contact angle of ≈130° and a hysteresis of 25°. Thus, in this case, the nanowire network exhibits a higher water droplet adhesion than a fresh rose petal.

This observed phenomenon is very similar to the rose‐petal effect. As shown in Figure 7 d–f, the rose petal surface exhibits both high contact angles and high contact angle hysteresis. There are certain similarities between the structural geometry of the rose petal and of our NWNWs. The rose petal consists of a periodic array of micro pillars, which are up to 25 µm high, with sub‐micro sized folds (0.5–1 µm) on their surface. The pillars have angles of up to 50° with slight variations of the inclination angle. It offers, therefore, a special combination of surface micro‐ and nanostructures.^[^ ^31^, ^33^, ^36^ ^]^ Meanwhile, our highly porous network structures exhibit total heights of ≈20–30 µm and nanowire inclinations of 45° with a certain inclination distribution due to the bending of the nanowires mentioned above, and displayed in Figure 2 a. The interwire distance for the highly porous networks is ≈0.5–0.8 µm, resembling the size of the rose petals pillar folds.

There were several extensive theoretical research studies about the origin of the rose‐petal effect.^[^ ^30^ ^]^ Traditionally, Cassie–Baxter theory is applied to describe situations when the liquid cannot wet the surface structure, and some air pockets are formed and trapped inside, which makes the surface hydrophobic with a high contact angle. On the petal surface, due to the high structural porosity, air pockets are trapped beneath the water droplet, so water cannot fulfill the whole nanostructure, resulting in a high contact angle and high adhesion.^[^ ^30^, ^33^ ^]^ On the other hand, Gao^[^ ^36^ ^]^ and Jiang^[^ ^54^ ^]^ observed, that liquid can be in direct contact with the petal surface micropillars, exhibiting the Wenzel regime at the microscale. In summary, the rose‐petal effect is attributed to a combined state between the Wenzel and Cassie–Baxter wetting regimes.

More generally, it has been previously discussed in literature,^[^ ^51^, ^53^, ^55^ ^]^ that complex surfaces can be defined by multiple roughness levels. In the case of the NWNWs investigated in this work, the first level is defined by the wires and their interwire distance (quantified as porosity in this work), while the second level is defined by the intrinsic hydrophilicity of the material. Consistently, our results indicate that the wettability of the NWNW is influenced by the different factors of its complex structure and thus describing the behavior of NWNWs requires a combination of Wenzel, Cassie–Baxter, and impregnating Cassie–Baxter theories.

It is important to mention that the Au nanowire networks exhibit a very stable performance. Thanks to the chemical stability and non‐oxidative nature of gold, a high contact angle and high adhesion are exhibited after one year of storage in the air. This long‐term stability makes this special nanostructure an ideal candidate for potential applications, such as for lab‐on‐a‐chip devices or microelectronics, where they need controlled movement or positioning of tiny liquid droplets on superhydrophobic surfaces.^[^ ^39^ ^]^

## 3 Conclusion

We demonstrated that 3D Au NWNWs exhibit a clear porosity‐dependent wettability change, as a function of their geometrical parameters. Thanks to a systematic and independent variation of nanowire density and diameter, we could show that the NWNWs with low‐porosity (<60%) show hydrophilicity (CA = 20°–50°), while the medium‐porosity samples from 60% to 80% show super‐hydrophilicity (CA < 10°), where imbibition effect plays a major role. The NWNWs with porosity higher than 95% exhibit very high contact angles (120°–130°) and high adhesion of water droplets to the sample surface, which revealed three major aspects of the rose‐petal effect: high contact angle, high adhesion, and high contact angle hysteresis. Our results also demonstrated that the developed tailored porous Au material exhibits long‐term stability, controlled wetting states, and good reproducibility. This innovative method offers a new way for enhanced control over surface properties, opening up new possibilities for various applications where tailored wetting behavior is desirable, such as microelectronics, optical devices, and energy harvesting devices.^[^ ^16^, ^56^, ^57^ ^]^

## 4 Experimental Section

### Sample Fabrication

The Au nanowire networks (NWNW) were fabricated by electrodeposition in ion track‐etched PC templates, which included four steps as shown in Figure 7. First, 30 µm polycarbonate (PC) foils (Makrofol N, Bayer AG) were irradiated with ≈2 GeV Au ion beam from four different directions, each time with a fluence between 1 and 5 × 10^8^ ions cm^−2^ at the UNILAC linear accelerator of the GSI Helmholtz Centre for Heavy Ion Research in Darmstadt (Germany). Each ion generates a very localized cylindrical damage zone, called an ion track. By chemical etching, the ion tracks were selectively etched and converted into open channels. The etching process was conducted at 50 °C, in a 6 m NaOH solution, with an etching rate of ≈25 nm min^−1^. This allows us to obtain templates with interconnected nanochannels with controlled channel sizes between 20 and 2000 nm.^[^ ^58^, ^59^, ^60^, ^61^ ^]^ One side of the template was covered by a thin sputtered Au layer (Edwards Sputter Coater S150B, pressure 10^−1^ torr, current 30 mA, 200 s), then strengthened by galvanostatic electrodeposition of Au at room temperature, applying 2 mAcm^−2^ current density using a commercial gold sulfite electrolyte (AuSF, 15 gL^−1^ Au, METAKEM). This back layer fully closes the nanochannels from one side and acts as a substrate for nanowire growth. The electrodeposition of the nanowires was performed potentiostatically at 60 °C, using a cyanide‐based alkaline electrolyte, which contained 50 mM KAu(CN)_2_, 250 mM Na_2_CO_3_, and 1 vol% Dowfax 2A1 surfactant, applying a potential of −0.9 V versus Ag/AgCl reference electrode (Sensortechnik Meinsberg GmbH, sat. KCl). The electrodeposition times ranged between 3 and 5 h. Afterward, the PC template was dissolved by immersing the sample in sequential solutions of dichloromethane.

### Sample Characterization

The geometry and morphology of the nanowire networks were characterized by high‐resolution scanning electron microscopy (HRSEM, Zeiss Gemini 500 field emission microscope).

Contact angle measurements of the nanowire networks were carried out using an optical tensiometer (Biolin Scientific Theta Lite), using the static sessile drop method. Droplets of deionized water (Milli‐Q Direct 8) of 4 and 15 µL volume were placed on the sample surface. The photographs were taken at five different spots on the surface for each sample.

For the tilting contact angle measurements, droplets were formed at the pipette tip, and then placed on the sample surface. The advancing angle and receding angle were measured at tilted 0, 15, 30, 45, 60, 75, and 90 degrees, with 4 and 15 µL droplets.

Optical images were taken by Keyence digital microscope VHX‐7000.

Surface analysis of the Au NWNWs was performed by X‐ray photoelectron spectroscopy (see supporting information, Figure S3, Supporting Information)

### Statistical Analysis

For two series of samples, the nanowire density was calculated from the nanochannel densities from the corresponding SEM images of the polymer templates, as shown in Figure S2 (Supporting Information). For each sample, 100 nanowires’ diameter was measured from their SEM images, and then the mean values and standard deviations were calculated.

For the contact angle measurement, each sample was measured freshly after fabrication and SEM analysis. For every measurement, after the droplet was placed on the sample surface, and in a steady state, a video was recorded for 5 s, the high‐speed camera can record 20 frames each second, and from each frame, one contact angle value is measured automatically from the software, then the average value could be calculated. This process was done five times for each sample at different locations, the mean value and standard deviation value were calculated, as shown in the plotted figures as well as in Table S1 (Supporting Information).

## Conflict of Interest

The authors declare no conflict of interest.

## Supporting information



Supporting Information

Supplemental Video 1

## Data Availability

The data that support the findings of this study are available from the corresponding author upon reasonable request.
