# Supplementary material for: 3D Gold Nanowire Networks with Tailorable Surface Wetting State: From Rose‐Petal Effect to Super‐Hydrophilicity
Source: Small. 2025 Apr 14;21(22):2411971. doi: 10.1002/smll.202411971 (PMC12138854; doi:10.1002/smll.202411971)
Supplement: Supplementary file 1 — Supporting Information [file SMLL-21-2411971-s001.docx]

Supporting Information

**3D Gold Nanowire Networks with Tailorable Surface Wetting State: from Rose-Petal Effect to Super-Hydrophilicity**

**Detailed demonstration of the 3D nanowire network structure**

The 3D nanowire network structures are primarily defined by the geometry of the nanochannels inside the polymer template. Figure 1 and Figure S1 illustrate how the irradiation step defines the initial 45° orientation and the density of the nanochannels.


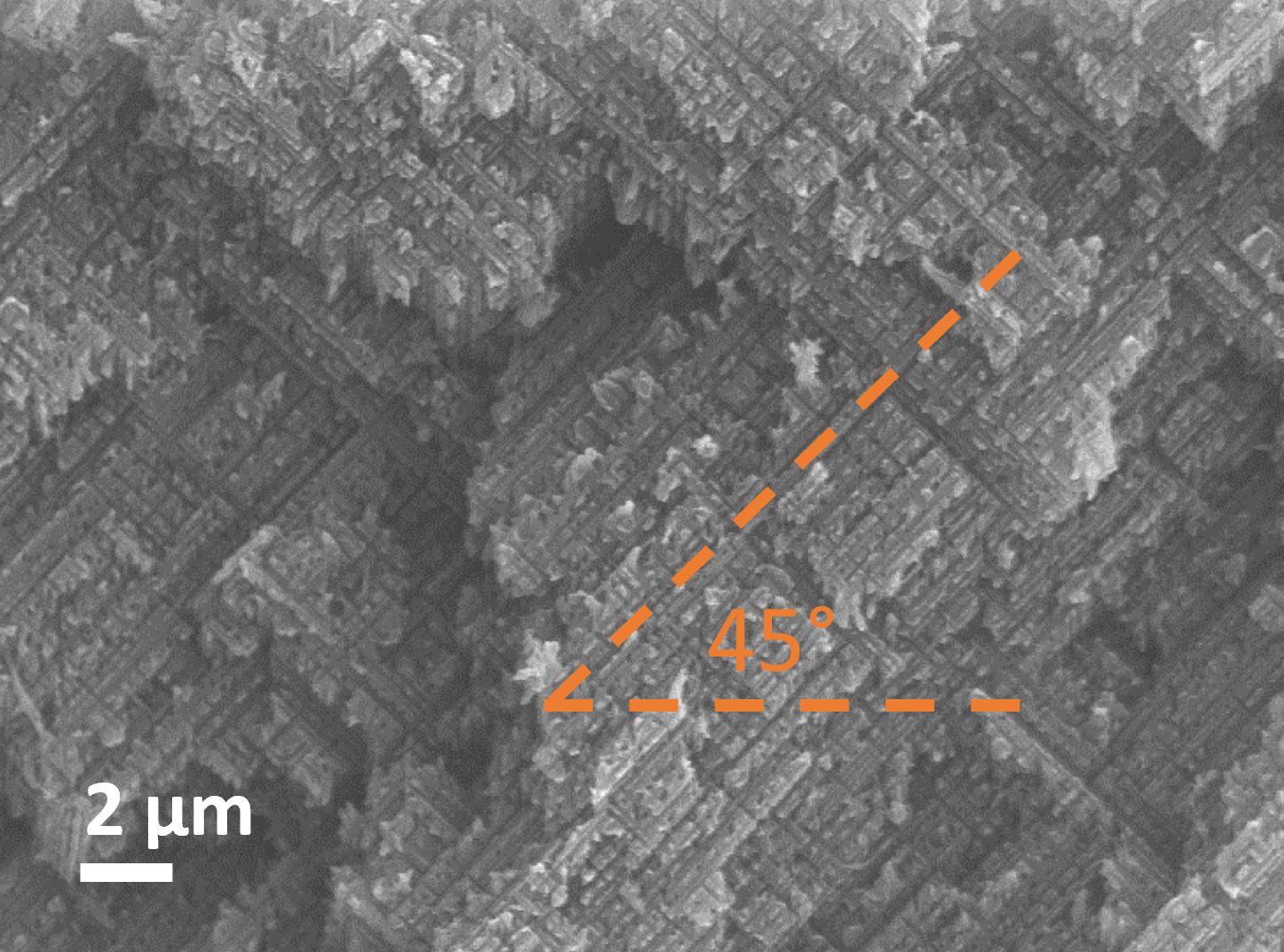


**Figure S1**. SEM image of a polymer template cross-section with irradiations from four different directions, with two directions perpendicular to the observing direction.

**Determination of the porosity of the NWNWs**

The structural porosity is calculated based on the geometrical parameters:

$$\Phi= \frac{V_{nw}}{V_{tot}}=\frac{\pi\times r_{nw}^{2}{\times L}_{nw}\times f_{nw}}{h}$$

$V_{nw}$ is the volume of all the nanowires, $V_{tot}$ is the total volume of the space, $\pi=3.14159$ is the ratio of a circle's circumference to its diameter, $r_{nw}$ is the radius of the nanowire, *h* is the height of the network structure, while $L_{nw}$ is the length of the nanowire, which is 1.41 times longer than *h*, because the wires are 45° tilted. The $f_{nw}$ is the density of nanowires.

**Nanowire network template density measurement from SEM images**

The nanowire density was defined as the nanochannel density from the PC templates after heavy ion irradiation and chemical etching, which is calculated from 10 different SEM images taken from the smooth side of the polymer surface, representative SEM images are shown in Figure S2. The template density defines the nanowire density in the network structure.


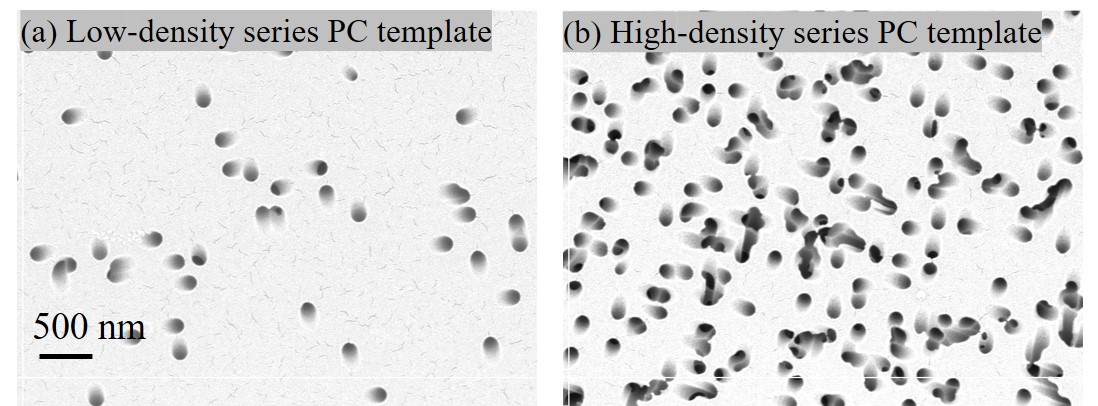


**Figure S2**. SEM images of etched PC templates with pore density of (a) $\left( 2.27\pm0.33 \right)\times{10}^{8}$ cm^-2^ (low-density) and (b) $\left( 1.26\pm0.09 \right)\times{10}^{9}$ cm^-2^ (high-density).

**Surface composition X-ray photoelectron spectroscopy (XPS) analysis of Au nanowire networks**

Figure S3 shows a representative XPS spectrum measured on a Au NWNW samples with diameter 150 nm and density 4*10^8^ cm^-2^. The spectrum reveals the main peaks (Au 4f, Au 4d expected for the Au NWNWs as well as three smaller peaks (C1s, O1s, and Na 1s) that can be attributed to small amounts of C, O, and Na residues^[1–4]^. The C and O is commonly seen when sample is exposed to air^[5,6]^, while the Na may originate from the nanowire fabrication process, as the electrolyte employed for the NW synthesis contains Na_2_CO_3_.


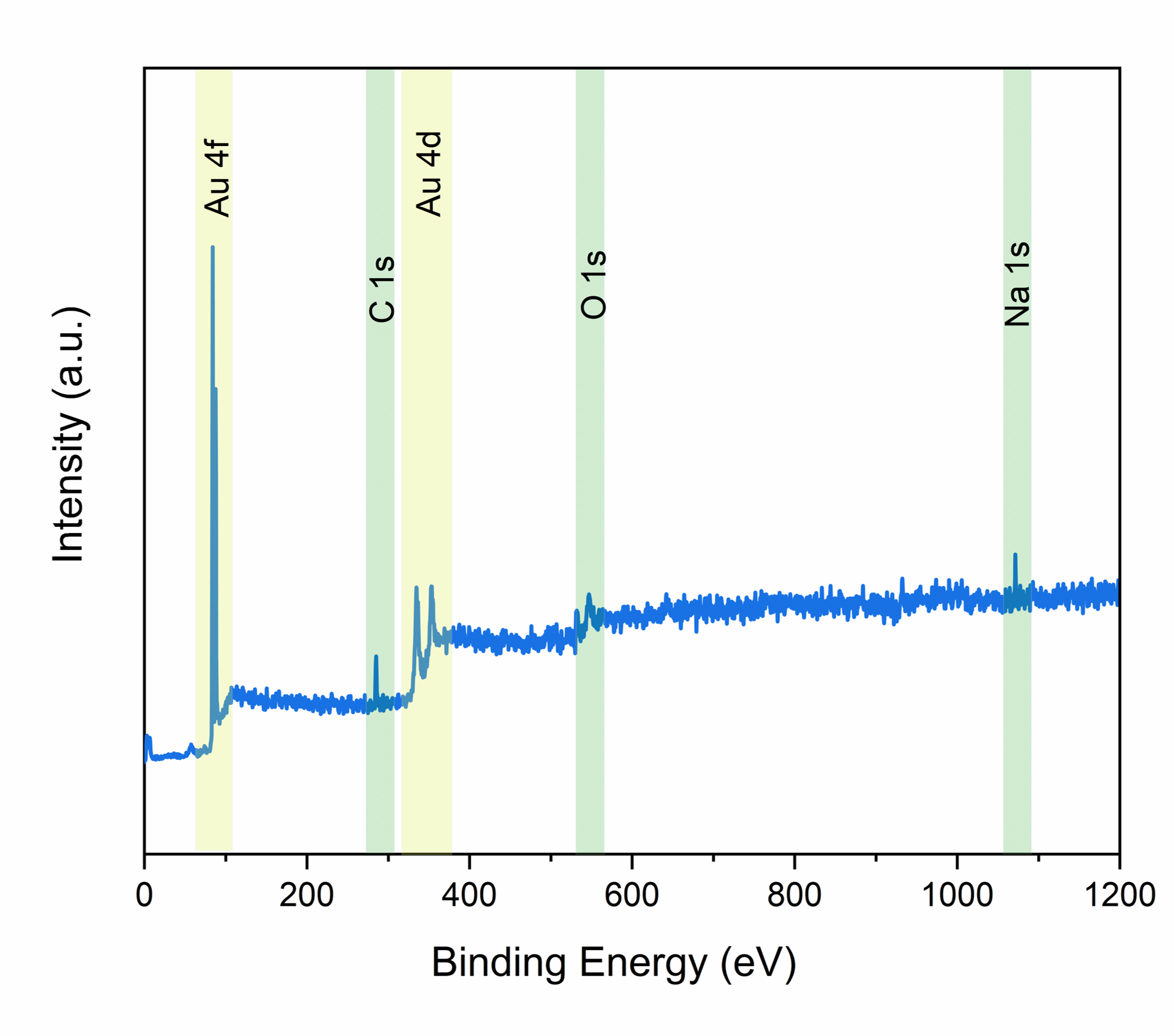


**Figure S3**. XPS spectrum of a gold nanowire network sample.

**Surface wetting photos**

For the hydrophobic samples, we did not observe any spreading of the droplet and there is no wetted area around the droplet (Figure S4).

Snapshots from the recorded video are presented in order to visualize the droplet spreading dynamic. On the hydrophilic samples, we observed a quick spreading of the droplet, and identified a wetted ring around the spread droplet (Figure S5, S6), then it stayed steady for several minutes.


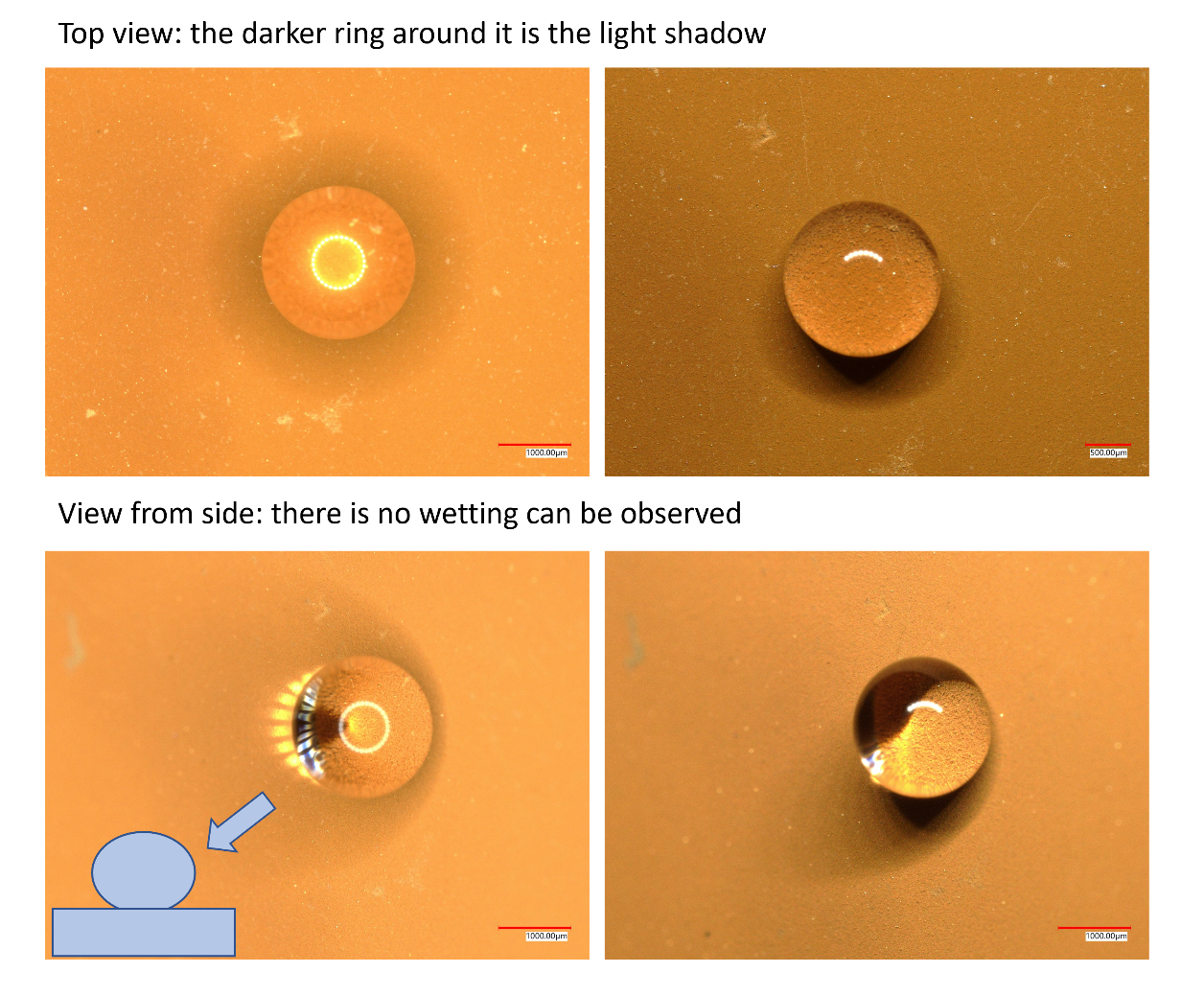


**Figure S4**. Photos of a 5 µL droplet on top of a hydrophobic nanowire network sample by Keyence optical microscope, scale bar is 1 mm. Nanowire diameter 104 nm, density 2.27*10^8^ cm^-2^, with porosity of 97%.


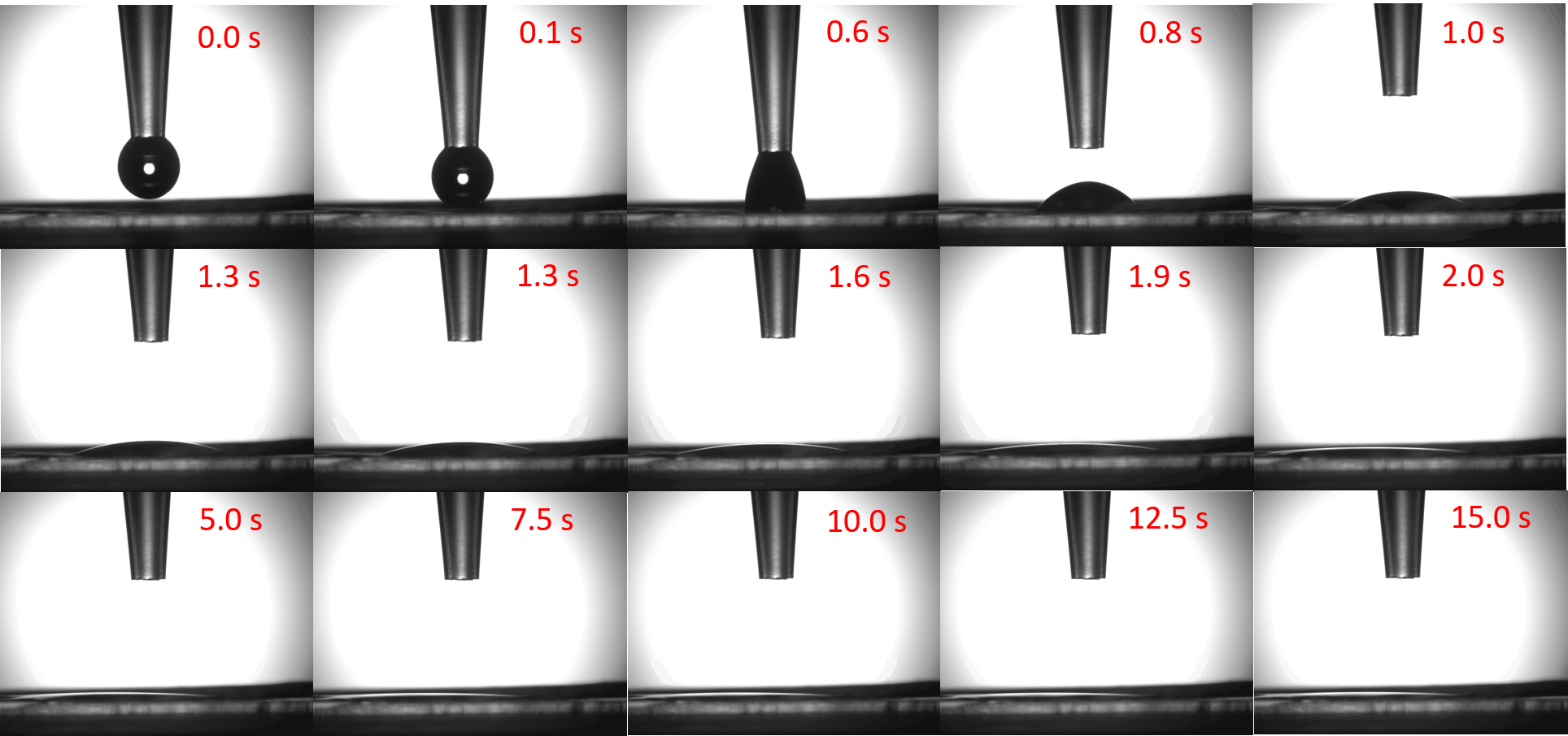


**Figure S5**. Photos of the sessile drop contact angle measurement by the high-speed camera on the Biolin Scientific Theta-lite tensiometer. On a hydrophilic NWNW sample, with nanowire diameter 106 nm, density 1.26*10^9^ cm^-2^, and porosity of 80%.


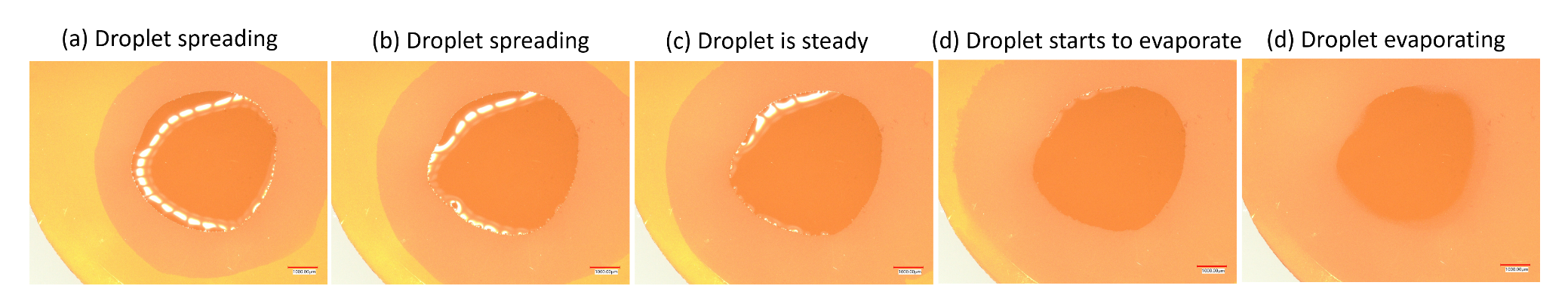


**Figure S6**. Photos of a 5 µL droplet on top of a hydrophilic nanowire network sample by Keyence optical microscope, a wetted ring around the droplet can be observed, scale bar is 1 mm. Nanowire diameter 120 nm, density 1.26*10^9^ cm^-2^, with porosity of 74%.

**Table S1**. Measured contact angle data in Figure 2, 3, and 5

| Nanowire density / cm^-2^ | Average nanowire diameter / nm | Standard deviation | Calculated porosity/ % | Standard deviation | Average contact angle/ ° | Standard deviation |
| --- | --- | --- | --- | --- | --- | --- |
| 2.27E+08 | 66.5 | 6.8 | 98.6 | 0.6 | 127.4 | 3.2 |
|  | 66.9 | 4.8 | 98.6 | 0.5 | 135.1 | 5.4 |
|  | 100.2 | 12.7 | 96.8 | 1.5 | 129.3 | 6.4 |
|  | 103.5 | 9.5 | 96.6 | 1.3 | 127.5 | 13.8 |
|  | 108.5 | 5.5 | 96.2 | 1.0 | 123.4 | 6.6 |
|  | 123.8 | 12.8 | 95.1 | 1.9 | 114.5 | 3.5 |
|  | 142.9 | 17.2 | 93.4 | 2.9 | 129.2 | 9.4 |
|  | 169.8 | 7.6 | 90.7 | 2.3 | 114.7 | 9.6 |
|  | 204.4 | 10.1 | 86.6 | 3.5 | 117.8 | 5.9 |
|  | 210 | 8.2 | 85.8 | 3.4 | 103.7 | 19.9 |
|  | 210.2 | 5.4 | 85.8 | 2.9 | 85.9 | 20.3 |
| 1.26E+09 | 44.5 | 3.2 | 96.5 | 0.8 | 122.4 | 4.1 |
|  | 45.2 | 3.3 | 96.4 | 0.9 | 132.9 | 10.4 |
|  | 50.4 | 5.8 | 95.5 | 1.5 | 15.9 | 4.9 |
|  | 57.8 | 4.6 | 94.1 | 1.5 | 77.5 | 41.2 |
|  | 58.3 | 4.7 | 93.9 | 1.5 | 123.6 | 4.5 |
|  | 58.6 | 5 | 93.9 | 1.6 | 21.5 | 18.7 |
|  | 95.5 | 6 | 83.8 | 3.4 | 11.9 | 3.1 |
|  | 106.3 | 6.3 | 79.9 | 4.1 | 8.1 | 2.4 |
|  | 107.9 | 9.6 | 79.3 | 5.6 | 6.4 | 0.5 |
|  | 120.6 | 11.2 | 74.1 | 7.3 | 12.3 | 4.6 |
|  | 140.7 | 10.7 | 64.7 | 8.5 | 13.4 | 4.6 |
|  | 162 | 8.7 | 53.2 | 8.9 | 27.5 | 3.2 |
|  | 187.7 | 13.9 | 37.2 | 14.8 | 39.1 | 12.9 |
|  | 195.8 | 18.2 | 31.7 | 19.1 | 43.5 | 9.7 |
|  | 198.5 | 13 | 29.8 | 15.2 | 23.0 | 5.6 |
|  | 200.3 | 18.2 | 28.5 | 19.7 | 32.1 | 12.1 |

**Table S2**. Measured contact angle data in Figure 6(c)

| Tilting angle (°) | Contact angle (°) | Standard deviation | Contact angle (°) | Standard deviation | Contact angle (°) | Standard deviation | Contact angle (°) | Standard deviation |
| --- | --- | --- | --- | --- | --- | --- | --- | --- |
|  | 4 μL, Advancing angle |  | 4 μL, Receding angle |  | 15 μL, Advancing angle |  | 15 μL, Receding angle |  |
| 0 | 134.2 | 1.9 | 134.0 | 1.2 | 140.2 | 2.4 | 140.2 | 3.0 |
| 15 | 135.7 | 2.9 | 128.8 | 1.9 | 155.8 | 4.4 | 129.1 | 1.7 |
| 30 | 146.1 | 1.5 | 124.2 | 0.5 | 158.0 | 1.0 | 114.0 | 5.0 |
| 45 | 139.0 | 2.0 | 121.0 | 1.0 | 164.0 | 2.0 | 100.5 | 0.5 |
| 60 | 140.5 | 0.5 | 114.5 | 2.5 | 161.5 | 1.5 | 91.0 | 2.0 |
| 75 | 142.0 | 1.0 | 109.5 | 0.5 | 162.5 | 0.5 | 81.5 | 0.5 |
| 90 | 143.0 | 1.0 | 108.0 | 2.0 | 160.5 | 0.5 | 80.0 | 1.0 |

**Droplet sliding measurement**

We tested a series of hydrophobic samples with different geometrical parameters, under the same experimental conditions, and found a very similar behavior, two of the representative samples are shown in Figure S7.


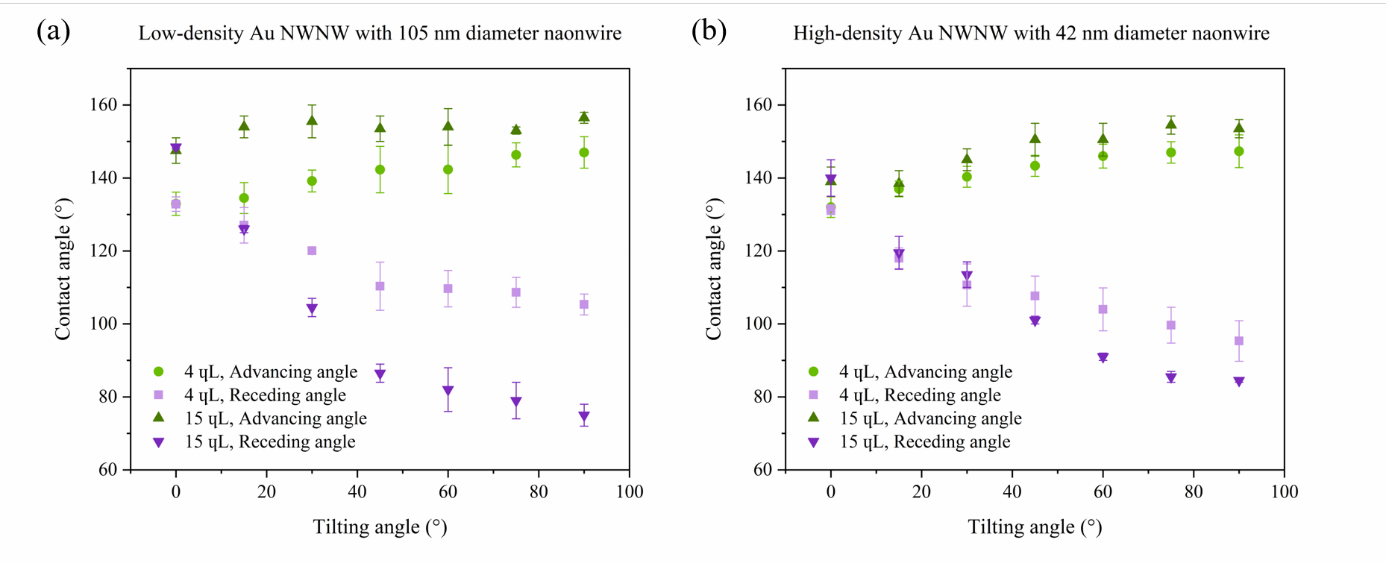


**Figure S7.** Plots of two-volume water droplets on the sample surface with advancing (green) and receding (purple) contact angles as a function of stage tilting angle, of (a) low-density Au NWNW with Ø 105 nm nanowire, and (b) high-density Au NWNW with Ø 42 nm nanowire.

**References**

[1] M. Daescu, A. Matea, C. Negrila, C. Serbschi, A. C. Ion, M. Baibarac, *Mol. 2020, Vol. 25, Page 4571* **2020**, *25*, 4571.

[2] C. S. Kam, T. L. Leung, F. Liu, A. B. Djurišić, M. H. Xie, W. K. Chan, Y. Zhou, K. Shih, *RSC Adv.* **2018**, *8*, 18355.

[3] A. Ghosh, S. Ghosh, G. M. Seshadhri, S. Ramaprabhu, *Sci. Reports 2019 91* **2019**, *9*, 1.

[4] T. C. Tudino, R. S. Nunes, D. Mandelli, W. A. Carvalho, *Front. Chem.* **2020**, *8*, DOI 10.3389/FCHEM.2020.00263.

[5] P. R. Norton, *Surf. Sci.* **1975**, *47*, 98.

[6] G. Greczynski, L. Hultman, *Prog. Mater. Sci.* **2020**, *107*, 100591.
